# Diffusion Tensor Imaging Evaluation of Corticospinal Tract Hyperintensity in Upper Motor Neuron-Predominant ALS Patients

**DOI:** 10.4061/2011/481745

**Published:** 2011-10-26

**Authors:** Venkateswaran Rajagopalan, Didier Allexandre, Guang H. Yue, Erik P. Pioro

**Affiliations:** ^1^Department of Biomedical Engineering, ND2, Lerner Research Institute, Cleveland Clinic, Cleveland, OH 44195, USA; ^2^Department of Physical Medicine and Rehabilitation S30, Neurological Institute S90, Cleveland Clinic, Cleveland, OH 44195, USA; ^3^Department of Neurology, S90, Neurological Institute, Cleveland Clinic, Cleveland, OH 44195, USA; ^4^Department of Neurosciences, NB2, Lerner Research Institute, Cleveland Clinic, Cleveland, OH 44195, USA

## Abstract

Amyotrophic lateral sclerosis (ALS) patients with predominant upper motor neuron (UMN) signs occasionally have hyperintensity of corticospinal tract (CST) on T2- and proton-density-(PD-) weighted brain images. Diffusion tensor imaging (DTI) was used to assess whether diffusion parameters along intracranial CST differ in presence or absence of hyperintensity and correspond to UMN dysfunction. DTI brain scans were acquired in 47 UMN-predominant ALS patients with (*n* = 21) or without (*n* = 26) CST hyperintensity and in 10 control subjects. Fractional anisotropy (FA), mean diffusivity (MD), axial diffusivity (AD), and radial diffusivity (RD) were measured in four regions of interests (ROIs) along CST. Abnormalities (*P* < 0.05) were observed in FA, AD, or RD in CST primarily at internal capsule (IC) level in ALS patients, especially those with CST hyperintensity. Clinical measures corresponded well with DTI changes at IC level. The IC abnormalities suggest a prominent axonopathy in UMN-predominant ALS and that tissue changes underlying CST hyperintensity have specific DTI changes, suggestive of unique axonal pathology.

## 1. Introduction

Amyotrophic lateral sclerosis (ALS) is a progressive degeneration of motor neurons in the brain and spinal cord whose cause is unknown [1]. It is also unclear whether the motor neuron degeneration begins in the perikaryon (cell body) as a neuronopathy and proceeds anterogradely, or along the axon as an axonopathy and proceeds retrogradely. Brain motor neurons, mostly in the primary motor cortex (upper motor neurons), project their axons caudally along the corticospinal tract (CST) through the brainstem to the spinal cord where they synapse onto anterior horn cells (lower motor neurons). Progressive degeneration of these two motor neuron pools results in ALS with upper motor neuron (UMN) signs (hypertonicity, hyperreflexia, and pathologic reflexes) and lower motor neuron (LMN) signs (muscle fasciculations, atrophy, and weakness) both being required for diagnosis [1]. A certain percentage of ALS patients present with UMN-predominant disease, and few or no clinically detectable LMN signs. By El Escorial criteria, they are usually initially categorized as possible or, at most, probable with laboratory support ALS because of the limited extent of LMN findings [2]. As disease progresses, their LMN dysfunction may remain either limited in degree or become more extensive.

Independent of this observation, a relatively small percentage of ALS patients display bilateral hyperintensity of the corticospinal tract (CST) on T2- and proton-density-(PD-) weighted MRI sequences [1]. Such hyperintensity can be seen anywhere along the rostrocaudal extent of the intracranial CST extent from just beneath the primary motor cortex, corona radiata, and centrum semiovale, but it tends to be most prominent in the posterior limb of the internal capsule and cerebral peduncles. A single study in 1994 of three ALS patients with CST hyperintensity who at postmortem had demyelination and Wallerian degeneration in the posterior limb of the internal capsule (IC) [3]. Unexpectedly, we have identified other ALS patients with similar UMN-predominant clinical presentations but without CST hyperintensity. The reason for this variability between presence or absence of CST hyperintensity and the essentially identical UMN-predominant clinical features is unclear but may reflect a different location of maximal pathology in the nonhyperintense group CST (e.g., in spinal cord), or intracranial pathologies with distinct axonal/periaxonal characteristics.

Previous brain MRI reports of hyperintensity of CST in ALS have been qualitative using conventional T2- and PD-weighted [1] as well as FLAIR-weighted [1] sequences. Because such qualitative approaches depend on several factors, including quality of MR image, limits of visual detectability, and the interpreter's experience, more quantitative MR methods would validate these findings and potentially provide insights into pathogenesis of the CST hyperintensity. Diffusion tensor imaging (DTI) is a relatively recently described modality based on the freedom with which water molecules (protons) move randomly within tissue, with intact myelinated axons providing the greatest restriction to movement between them (high anisotropy) [4]. Because DTI signal is dependent on microscopic-level events, it has the potential of detecting tissue pathology at/near submacroscopic levels, even before the changes are visible by conventional MRI. Identifying pathologic changes noninvasively at early stages would shorten time to diagnosis and allow timely therapeutic intervention. Because the CST and other subcortical white matter in ALS brain are usually normal in appearance by conventional MRI, DTI should still be able to detect abnormalities. Furthermore, the organization of subcortical myelinated motor axons as compact parallel bundles (e.g., CST) can be ideally interrogated by DTI because its metrics will reflect whether they are intact (healthy) or disrupted (degenerating).

Therefore, the main goal of this study was to use DTI obtained at 1.5T as part of routine clinical neuroimaging of ALS patients with predominant UMN signs for quantitative evaluation of the intracranial CST, which in one group was hyperintense on T2- and PD-weighted sequences. This would allow us to determine where abnormalities in DTI metrics occur along the CST in our UMN-predominant ALS patients, and whether quantitative differences are detectable corresponding to the qualitative presence or absence of CST hyperintensity. We hypothesized that motor neuron degeneration in UMN-predominant ALS is anterograde, arising primarily in the perikaryon as a neuronopathy. If this is the case, DTI metrics would be expected to be more abnormal at more rostral levels of the CST intracranially. Second, we hypothesized that CST hyperintensity in ALS brain reflects a more extreme case of such anterograde degeneration that has unique tissue characteristics detectable by DTI. 

By testing these hypotheses, we hope to have a better understanding of CST degeneration in UMN-predominant ALS patients and to identify quantitative DTI measures which may be useful in objectively differentiating disease subtypes.

## 2. Methods

### 2.1. Demographics

1.5T MRI data obtained as part of clinical neuroimaging evaluation was approved by the Institutional Review Board at the Cleveland Clinic to be stored and analyzed as deidentified images after patients had provided verbal consent. DTI data were analyzed in the following patient groups: (1) 10 neurological controls (7 men, 3 women) aged 51.1 ± 7.3 years (mean ± SD, range 28–80 years), (2) 21 UMN-predominant ALS patients (14 men, 7 women) with CST hyperintensity on T2/PD-weighted images (CST+) aged 52.3 ± 11.02 years (range 32–75 years), and (3) 26 UMN-predominant ALS patients (14 men, 12 women) without CST hyperintensity identified on T2/PD-weighted images (CST−) aged 59.5 ± 12.1 years (range 32–76 years). UMN-predominant ALS patients were defined as those with either no lower motor neuron signs or, if present, then restricted to only one neuraxial level (bulbar, cervical, or lumbosacral) at the time of MRI. Duration of symptoms prior to MRI in the CST+ group was 9.6 ± 5.5 months (mean ± SD) and in the CST− group was 36.4 ± 44.2 months; the large standard deviation is due to two outlier values in the latter group of 148 and 180 months. El Escorial diagnostic criteria [2] assigned to each patient after their clinical evaluation were converted to a numeric form as follows: possible = 1, probable with lab support = 2, probable = 3, and definite = 4. This El Escorial criteria score in the CST+ group was 1.81 ± 0.98 (mean ± SD) and in the CST− group was 1.37 ± 0.82.

### 2.2. DTI Data Acquisition

DTI data were obtained on a 1.5T system (Siemens Symphony, Erlangen, Germany) using echo planar imaging (EPI) sequence along 12 diffusion-weighted (b = 1000 s/mm^2^) directions and one b_0_ = 0 s/mm^2^. Imaging parameters were 30 slices, 4 mm thick, with 1.9 × 1.9 mm in-plane resolution; pulse sequence parameters were TR = 6000 ms, TE = 121 ms, EPI factor = 128, number of averages = 6, and scan time = 7.54 minutes. Gradient-echo field map images were acquired to correct for geometrical distortion caused by susceptibility artifacts. Field map imaging parameters were 30 slices, 4 mm thick, 4 mm slice gap, TR = 500 msec, TEs = 6.11, and 10.87 msec. T2-and PD-weighted images were obtained using dual-echo FSE sequence whose imaging parameters were number of slices = 40, contiguous, slice thickness = 4 mm, and in-plane resolution = 0.9 × 0.9 mm; pulse sequence parameters were repetition time (TR) = 3900 ms, TE = 26 ms and 104 ms, echo train length or turbo factor = 7, and number of averages = 1; total scan time = 3.5 minutes. 

### 2.3. Data Processing

DTI images were first corrected for susceptibility artifacts and eddy current distortions using FSL FUGUE and eddy current distortion correction algorithm in FSL (http://www.fmrib.ox.ac.uk/fsl/) [5–7]. The b-matrix was rotated in order to preserve the correct orientation information after eddy current and oblique angle corrections [8, 9]. The above preprocessed DTI images were then processed using DTI Studio open software (https://www.mristudio.org/) [10]. DTI matrix for each voxel element was calculated based on multivariate linear least square fit. The tensor matrix was then diagonalized to derive principal eigenvalues and eigen vectors. Maps of diffusion metrics, namely, fractional anisotropy (FA), mean diffusivity (MD mm^2^s^−1^), axial diffusivity (AD, *λ*_||_ mm^2^s^−1^), and radial diffusivity (RD, *λ*_⊥_ mm^2^s^−1^), were obtained. Virtual nerve fibers were reconstructed using the fiber assignment by continuous tracking (FACT) algorithm [10], described in detail elsewhere [11]. Fiber tracking parameters were initiated from every voxel with FA = 0 [12], threshold for termination 0.2 [12], and a bending angle of 41°. 

**Figure 1:**
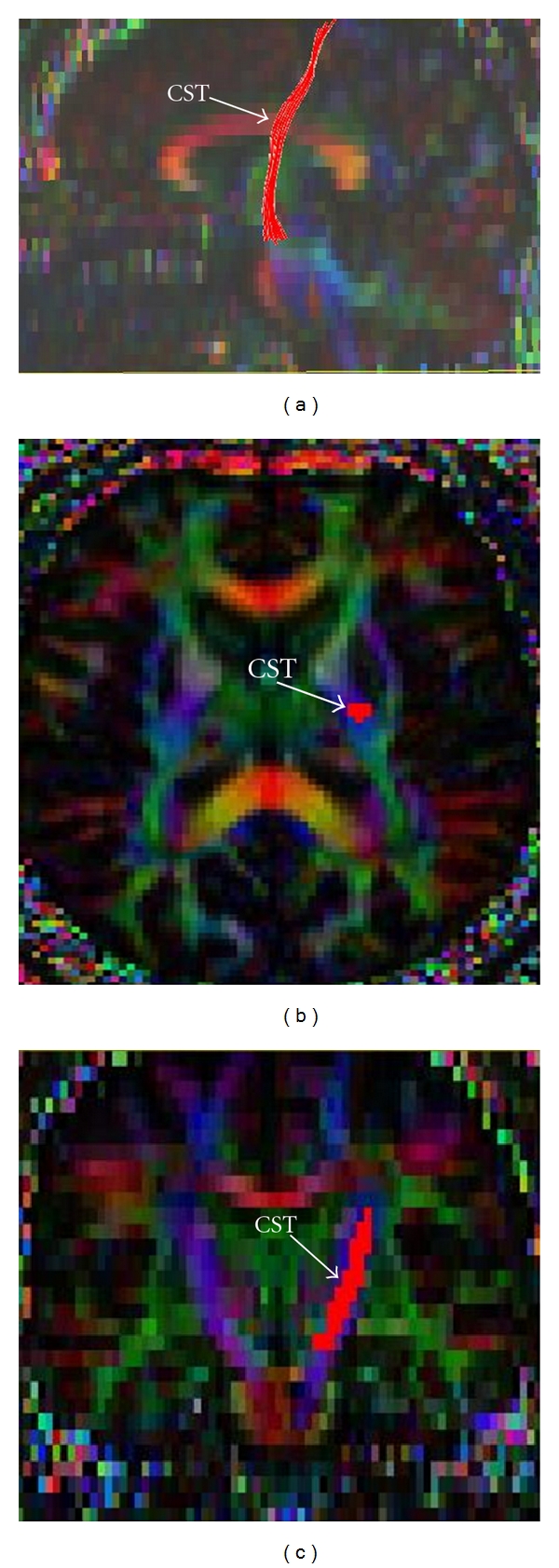
Corticospinal tract (CST) reconstructed using tractography superimposed on a subject's own FA color map in (a) sagittal, (b) axial, and (c) coronal planes.

After above steps, both control and ALS patients' CST fiber tracts on both left and right sides were reconstructed (“tractography”) following Wakana et al.'s method [12] by placing first ROI caudally in cerebral peduncle and the second rostrally just below the primary motor cortex. Four regions of interest (ROIs) were identified *a priori *at specific levels along the CST (using b_0_ = 0 and FA images) for DTI measures, including cerebral peduncle (CP), posterior limb of the internal capsule (IC), and centrum semiovale at top of lateral ventricle (LV) and subjacent to primary motor cortex (MC). DTI metrics of FA, MD, AD, and RD were measured in each ROI after superimposing each subject's own CST tractography mask on their DTI maps. Values were then compared between ALS patients and controls and also between the patient groups.  Figure 1 shows the left CST mask superimposed on a subject's FA color map.

Statistical comparisons for each of the DTI metrics across control and patient groups were carried out using SPSS 16.0 (SPSS Inc., Chicago, Ill, USA). Based on data meeting the assumptions of ANOVA, one of the following statistical methods was used with a significance level of *P* < 0.05. One-way ANOVA followed by Tukey's post hoc test was carried out when the assumptions of both normality and equal variance were met. If the equal variance assumption was violated, the Welch ANOVA followed by the Dunnett T3 post hoc test was employed. When both normality and equal variance assumptions were violated, the Kruskal-Wallis nonparametric approach followed by the Bonferroni correction of the Mann-Whitney test was used. Clinical parameter of symptom duration prior to MRI was correlated with DTI metrics in all the 4 ROIs along the CST using Spearman's correlation method after correcting for multiple comparisons using false discovery rate (FDR).

Because abnormalities of DTI values along the CST may represent degeneration or related pathology, we assessed whether such metrics correlated with pathologic UMN signs (spasticity, hyperreflexia, and pathologic reflexes), as identified by neurologic examination performed at clinical evaluation near/at time of MRI. Because CST fibers decussate below the lowest ROI analyzed for DTI metrics (at the cervicomedullary junction), the intracranial CST corresponds to the contralateral side of body reflecting UMN signs. In order to detect a side-to-side asymmetry in CST DTI values which may correspond to asymmetry of UMN signs, ratios of side-to-side DTI values (right to left) for FA, MD, AD, and RD in all the 4 ROIs along CST were calculated in control and in both the ALS patient groups. For this analysis, all ALS patients (both CST+ and CST− groups) were categorized by their UMN-predominant body signs being primarily right sided or left sided, based on their clinical evaluation at time of MRI. Patients with predominantly right body UMN signs should have more abnormal left CST DTI metrics, and vice versa. For example, a patient with mostly right body UMN signs would have lower FA and AD values in the left CST, so right to left ratio would be greater than or equal to 1, whereas a patient with mostly left body UMN signs would have lower FA and AD values in the right CST, so right to left ratio would be less than or equal 1. Because an inherent asymmetry was observed between right and left CST DTI values even in controls, the mean right to left ratio from control values was used as a threshold to estimate the number of ALS patients above (with more right body UMN signs) or below (with more left body UMN signs) it. A percentage value was then calculated of patients whose ratios were abnormal DTI values in CST relative to control, which represents the degree of correspondence between abnormal DTI values in CST and the appropriate side of body with pathologic UMN signs, as shown in Table 1.

## 3. Results

**Figure 2:**
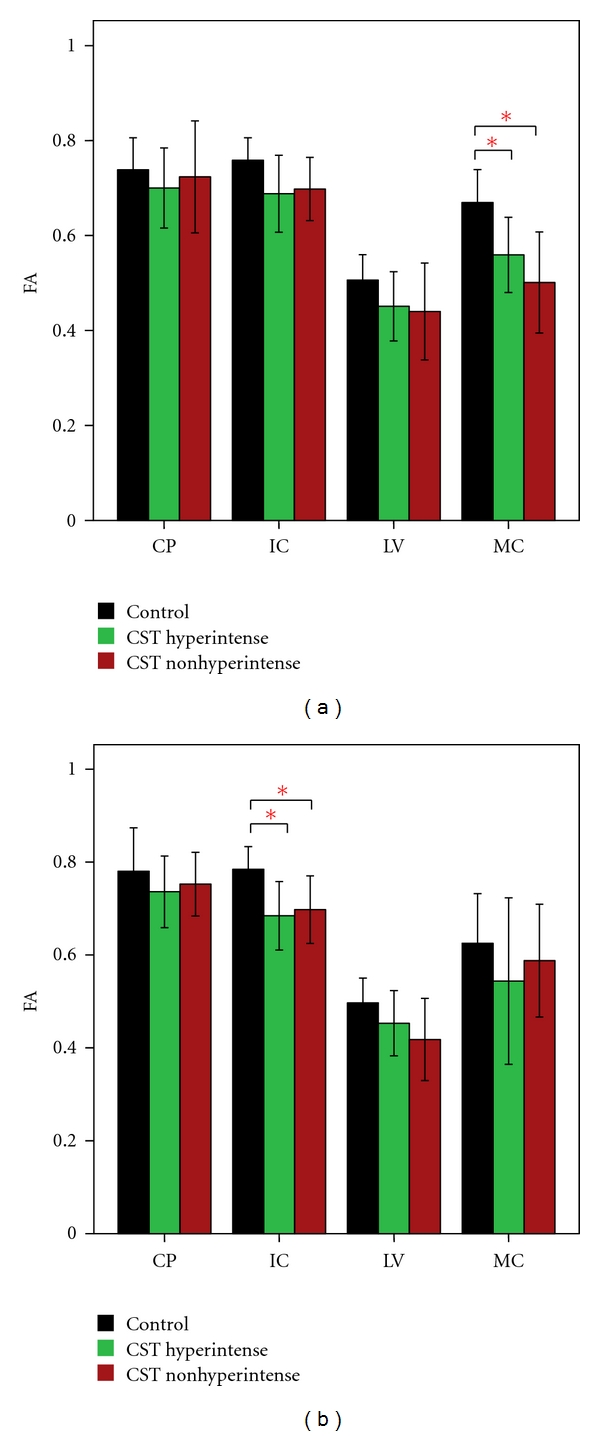
Fractional anisotropy (FA) at four CST levels on left (a) and right (b), revealing significantly lower values in right MC and left IC of CST hyperintense and CST nonhyperintense patients compared to controls. *P < 0.05. Key: CP, cerebral peduncle; IC, posterior limb of internal capsule, LV, centrum semiovale at top of lateral ventricle, MC, subcortical to motor cortex.

**Figure 3:**
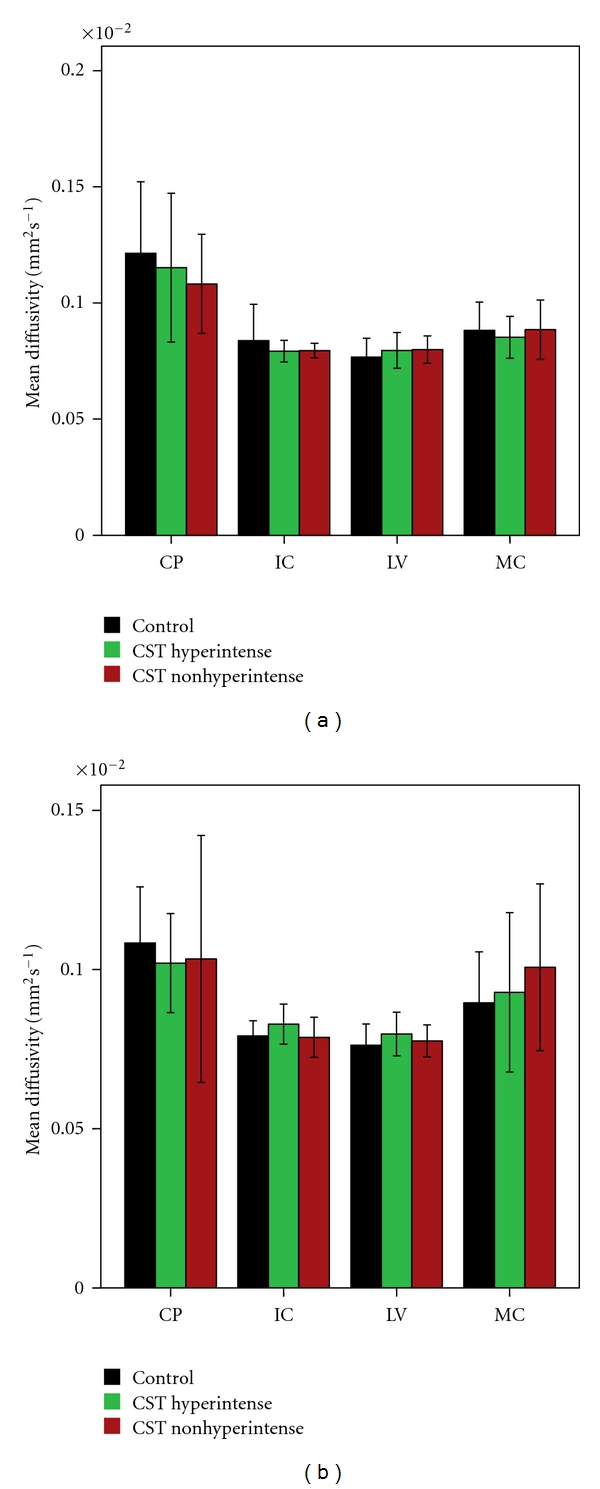
Mean diffusivity (MA) at four CST levels on left (a) and right (b), revealing no significant differences between groups. Key: CP, cerebral peduncle; IC, posterior limb of internal capsule, LV, centrum semiovale at top of lateral ventricle, MC, subcortical to motor cortex.

**Figure 4:**
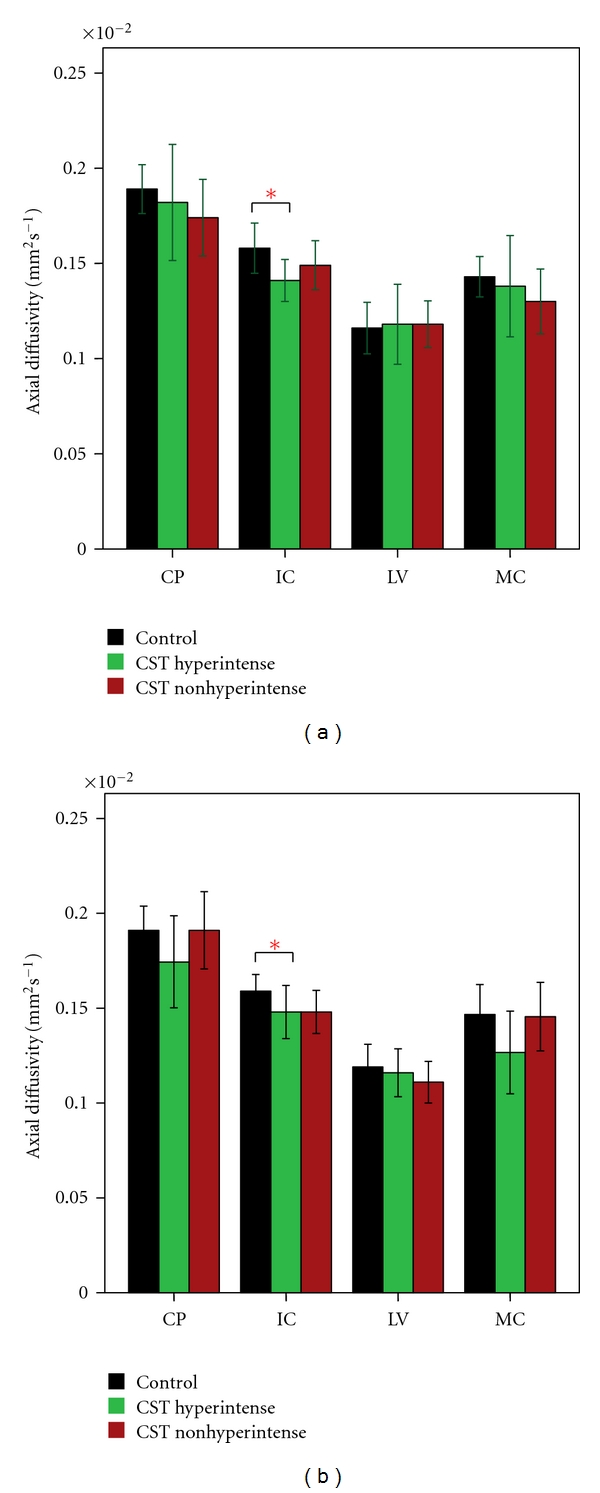
Axial diffusivity (AD) at four CST levels on left (a) and right (b), revealing significantly lower values at the IC level bilaterally in only CST hyperintense patients compared to controls. *P < 0.05. Key: CP, cerebral peduncle; IC, posterior limb of internal capsule, LV, centrum semiovale at top of lateral ventricle, MC, subcortical to motor cortex.

**Figure 5:**
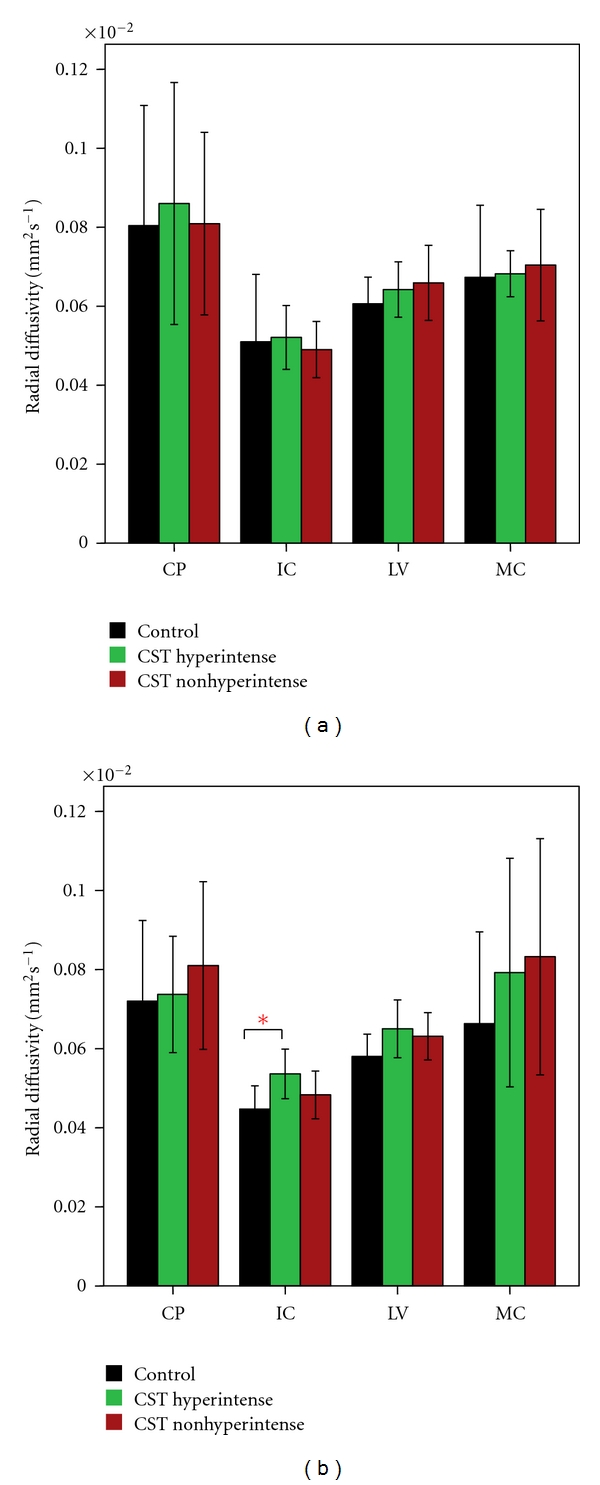
Radial diffusivity (RD) at four CST levels on left (a) and right (b), revealing significantly higher values at the IC level on the right in only CST hyperintense patients compared to controls. *P < 0.05. Key: CP, cerebral peduncle; IC, posterior limb of internal capsule, LV, centrum semiovale at top of lateral ventricle, MC, subcortical to motor cortex.

Mean FA, MD, AD, and RD values for each ROI along the CST and their significant differences (based on parametric and nonparametric tests depending on data meeting the assumptions of the test as described in Methods) between the 3 groups are given in Figures 2–5. FA showed significant difference between control and the ALS groups at right IC and left MC. However, no significant differences in FA values were observed between ALS subgroups in any of the 4 ROIs (Figure 2). MD values showed no significant differences in any of the 4 ROIs among the 3 groups (Figure 3). Axial and radial diffusivities showed significant differences only between control and ALS CST hyperintense groups and only at the level of IC (Figures 4 and 5).

**Figure 6:**
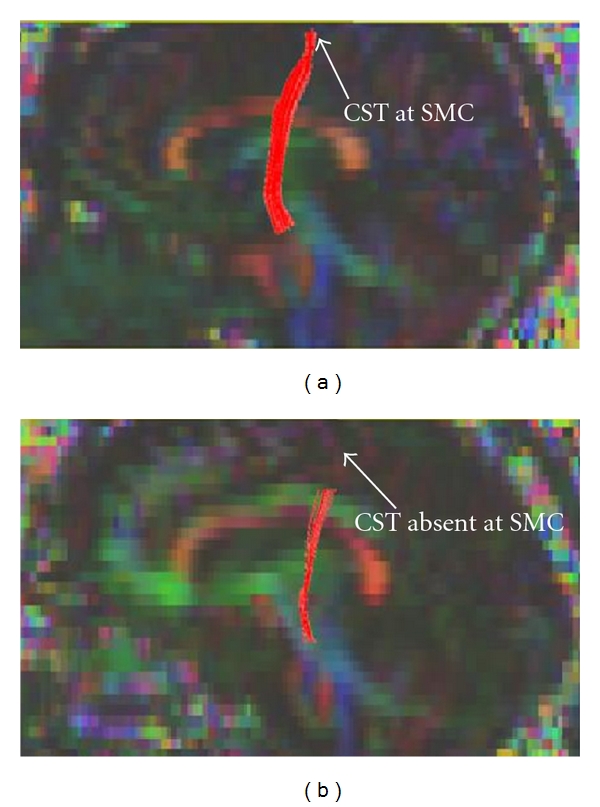
Sagittal views of FA color maps showing tractography-derived CST extending up to the submotor cortex (SMC) level in a control subject (a) but truncated in an UMN-predominant ALS patient with CST hyperintensity (b).

The CST was found to be truncated above the LV level, most prominently on the right compared to left in 12 subjects (of 21, 57%) in the hyperintense CST group; of these, CST was truncated on the right in 8 (of 12) and bilaterally in 4. Similarly, CST truncation was observed in 6 subjects in the nonhyperintense CST group (of 26, 23%); of these, truncation was on the right in all. Truncation of CST was not observed in any of the 10 control subjects. Figures 6(a) and 6(b) show CST tractography extending to the cortex in a typical control subject and a truncated CST in an ALS subject. Because the same DTI processing methodology was employed across all subject groups, this could not have accounted for the observed differences in CST truncation. We also investigated whether the absence of CST above the LV level in some patients may have lead to not detecting significantly different DTI metrics in the MC. Since tractography was used to identify the fibers to be measured, missing values at the MC level in patients with truncated CST could underestimate differences in DTI metrics between groups. To correct for this possibility, Mori's CST atlas was superimposed on the FA map, and values were measured in all 4 ROIs along the CST. This did not change the results, and still no significant differences were observed in FA values at the MC level of control and ALS groups even when identifying the CST using Mori's atlas. We further investigated whether the quality of DTI scans obtained at 1.5T, that is, resolution, anisotropic voxel dimension, and signal/noise ratio, contributed to the CST truncation problem. 3T DTI data were collected on another set of ALS patients with (in 1 patient) and without (5 patients) CST hyperintensity and in control subjects (5 patients). Imaging parameters of 3T data were almost identical to those of our 1.5T data except that at 3T voxels they were isotropic (2 × 2 × 2.5 mm) resulting in improved resolution, and signal to noise ratio was higher. CST tractography and analysis on 3T data were performed in an identical manner to the 1.5T data. Although the number of studies at 3T was limited, we found similar CST truncation in 2 of 5 ALS CST hyperintense patients and none in controls. 

 Symptom duration prior to MRI was significantly shorter (*P* < 0.0003) in ALS patients with CST hyperintensity (median = 13 months) than in those without CST hyperintensity (median = 31 months). No significant difference was found in El Escorial criteria scores between either of these two groups. No significant correlation was obtained between symptom duration prior to MRI and DTI metrics on any of the 4 ROIs along CST.

## 4. Discussion

Fractional anisotropy (FA) abnormalities seen in this study of CST at the level of internal capsule agree well with previous ALS studies [8, 13–17], although reaching significance only on the right. We also found significantly lower FA values in ALS patients at the subcortical motor cortex level on the left. The reason for this side-to-side variability is unclear. In general, FA values were reduced in both CST hyperintense (CST+) and CST nonhyperintense (CST−) ALS groups when compared to controls, but they were not significantly different between the ALS groups. The CST+ group did, however, have lower FA values than the CST− group. Of note, axial diffusivity (AD) and radial diffusivity (RD) values were significantly different at the IC level only between controls and the CST+ group and not the CST− group. Such differences in AD and RD abnormalities may reflect microanatomical pathologic differences in the CST in these two groups of ALS patients. It is known that FA may not be as reliable a measure as individual AD and RD, from which FA is calculated. Beaulieu [18] showed that FA value changes result from either decreased AD, increased RD, or changes in both. He also demonstrated in animal and human studies that AD is reflective of axonal integrity and RD is reflective of myelin integrity, with abnormalities in these DTI metrics representing their degeneration [18]. Such interpretations should, however, be interpreted with caution in the present study, as they are likely oversimplifications, and further studies would be required for confirmation. Nonetheless, the aforementioned suggests that AD and RD are more representative of microanatomical integrity than is FA. In general, FA, AD, and RD values were different between CST+ and CST− ALS groups but failed to reach statistical significance.

Lack of significant differences in MD values in all ROIs along the CST in this study generally agrees with other ALS studies [19, 20], although some DTI studies in ALS have found MD abnormalities at the IC level [21–23]. Reasons for this discrepancy may include pooling ALS patients of multiple clinical phenotypes into a single group in other studies, variable DTI data acquisition and processing parameters (our processing included oblique angle and susceptibility artifact corrections, not used in previous DTI studies in ALS), and use of ROI-based approach (our study used tractography of patient's own CST which is more accurate and reproducible). Furthermore, since ALS results in not only axonal/myelin degeneration (which would result in decreased FA and increased MD) but also gliosis [14, 24], the net diffusion revealed by MD may not be higher than control values. If this is the case, MD values would be expected to be higher in patients with shorter disease duration (and presumably less time for gliosis) than longer disease duration.

The fact that we found FA, AD, and RD abnormalities in the CST primarily at the IC level and not caudally at the CP level, and some abnormalities rostrally, suggests a degenerative process that may begin at midpoint along the subcortical myelinated motor axon. Although interpreting such DTI metrics as proof of axon/myelin loss is inaccurate, it indicates that CST microanatomy is sufficiently abnormal to result in perturbed water (proton) diffusion. Postmortem histopathologic studies of this region may elucidate the significance of these DTI findings. Supportive evidence that these DTI abnormalities at the IC level are related to the CST neurodegenerative process includes FA values showing 85% correspondence with left body UMN-predominant clinical signs and radial diffusivity as well as MD showing 94.4% correspondence with right body UMN-predominant clinical signs. Only correspondence with a 75% or greater threshold was chosen, to insure only a 25% correspondence occurred by chance. 

Furthermore, the preponderance of CST virtual fibers truncated at the subcortical motor cortex (MC) level in the CST+ over the CST− ALS patients (and never in control subjects) also suggests disease-specific changes rather than artifact in data acquisition or processing. How this CST virtual fiber truncation corresponds to axonal pathology is unclear and invites further study. Failure to detect significant changes in DTI metrics of the CST at the LV level could be due to effects of the superior longitudinal fasciculus whose fibers cross perpendicularly at that level. This region in control subjects also has decreased uniformity of fiber tracts (unpublished observations), consistent with crossing fibers (as measured by Westin's linear and planar indices [25]).

Duration of ALS symptoms prior to MRI was shorter in CST+ than in CST− group, which may reflect faster disease progression, although this is uncertain. In an early neuroimaging-pathologic study, Yagishtia et al. [3] found CST hyperintensity on MRI corresponds to demyelination and axon degeneration suggesting a different pathology from patients without CST hyperintensity. Other studies reported that ALS patients with CST hyperintensity had rapid clinical decline initially [1], shorter disease duration, and faster disease progression [26]. In a separate study evaluating 112 ALS patients over a 10-year period who had undergone at least one brain MRI, we found those with CST hyperintensity (*n* = 35) had significantly more rapid disease progression and shorter survival than those without CST hyperintensity (*n* = 77) [27]. It is therefore possible that ALS patients with CST hyperintensity have different underling pathology from those without CST hyperintensity, which results in faster disease progression. In the context of similar or slightly worse FA and AD/RD abnormalities at the IC level of the CST, this suggests a more rapidly evolving disease process in the CST+ group. Although correlations between duration of symptoms and individual DTI metrics at each of the ROIs along the CST did not reach statistical significance after correction for multiple comparisons (false discovery rate correction), there was a trend for correlation of FA, AD, and RD abnormality at IC and LV levels with disease duration when *P* < 0.05 is uncorrected for multiple comparisons. 

Although the DTI changes we observe between these ALS patient groups are small, they support differences visualized by qualitative T2/PD images and suggest they result from real microanatomic pathologic changes, such as inflammation, demyelination, axon loss, or gliosis [28]. However, detailed studies correlating CST changes detected by DTI and T2/PD with postmortem histopathology will be required to determine what causes the imaging abnormalities we have found.

To our knowledge this is the first study to use DTI to quantitatively evaluate the CST after its virtual reconstruction by tractography and classify UMN-predominant ALS patients into groups based on the presence or absence of CST hyperintensity. 

## 5. Conclusion 

DTI performed at 1.5T as part of routine clinical brain MR imaging demonstrates abnormalities in FA and related parameters predominantly at the IC level of the CST in UMN-predominant ALS patients compared to control subjects. This correlates with the appropriate body side showing more prominent UMN signs clinically. In addition, patients with CST hyperintensity have abnormalities of the AD and RD components of FA, while those without CST hyperintensity do not, suggesting differences in tract microanatomy. Furthermore, subcortical truncation of virtual CST fibers generated by tractography occurs more frequently in ALS patients who have CST hyperintensity than those who do not, again suggesting divergent pathology. Predominance of DTI abnormalities at the IC level and rostrally suggests an anterograde process arising from a neuronopathy of motor neurons forming the CST. Identification of these DTI abnormalities in UMN-predominant ALS patients from routine clinical scans demonstrates feasibility of acquiring useful quantitative information with a 1.5T magnet system. Future DTI studies at 3T with larger control subject group will confirm these findings.

## Figures and Tables

**Table 1:** Percent correspondence of abnormal CST DTI metrics with appropriate side of body showing more UMN signs.

	CP		IC		LV		PMC	
Body predominance of UMN signs	Left (%)	Right (%)	Left (%)	Right (%)	Left (%)	Right (%)	Left (%)	Right (%)
FA	50	33.00	**85**	28	68.40	66.60	16.60	45.50
AD (mm^2^s^−1^)	50	61.1	30	33	73.6	55.5	41.6	36.3
RD (mm^2^s^−1^)	50	55.5	30	**94.4**	52.6	55.5	58.3	45.4
MD (mm^2^s^−1^)	50	55.5	15	**94.4**	52.6	61.1	58.3	36.3

Key: AD: axial diffusivity, CP: cerebral peduncle, CST: corticospinal tract, FA: fractional anisotropy, IC: posterior limb of internal capsule, LV: centrum semiovale at top of lateral ventricle, MD: mean diffusivity, PMC: subjacent to primary motor cortex, RD: radial diffusivity, UMN: upper motor neuron.
